# CtGAP 2.0: enhanced genome assembly, strain resolution, and phylogenomics for *Chlamydia trachomatis*

**DOI:** 10.1128/mra.01470-25

**Published:** 2026-02-24

**Authors:** Olusola Olagoke, Timothy D. Read, Deborah Dean

**Affiliations:** 1 Department of Pediatrics, Division of Infectious Diseases and Global Health, University of California San Francisco School of Medicine 12224 , San Francisco, California, USA; 2 Benioff Center for Microbiome Medicine, University of California San Francisco 8785, San Francisco, California, USA; 3 Department of Medicine, Division of Infectious Diseases, Emory University School of Medicine 12239, Atlanta, Georgia, USA; 4 Department of Medicine, Division of Infectious Diseases and Global Health, University of California San Francisco School of Medicine 12224 , San Francisco, California, USA; 5 Department of Bioengineering, Joint Program, University of California San Francisco and University of California Berkeley 1438, Berkeley, California, USA; 6 Bixby Center for Global Reproductive Health, University of California San Francisco 8785, San Francisco, California, USA; University of Michigan, Ann Arbor, Michigan, USA

**Keywords:** *Chlamydia trachomatis*, genome assembly pipeline, genome analysis, phylogenomics, chromosome typing, plasmid typing

## Abstract

We present *Chlamydia trachomatis* Genome Assembly Pipeline (CtGAP) 2.0, an enhanced version of CtGAP, that provides a seamless installation process—eliminating the need for multiple standalone tools—and a streamlined command-line interface, introduces automatic assembly quality scoring and selection for downstream analyses, and integrates chromosome and plasmid typing along with phylogenomics.

## ANNOUNCEMENT

*Chlamydia trachomatis* (*Ct*) is a globally significant bacterial pathogen that is the leading cause of bacterial sexually transmitted diseases and infectious blindness worldwide (1). Previously, we introduced CtGAP (2), a pipeline that automates *Ct* genome assembly, genotyping, and phylogenomics. The tool accepts paired-end reads and performs quality control, host decontamination, and assembly via *de novo* or reference-guided approaches. Users may also choose to run both methods in parallel and manually inspect the output to select the best-performing assembly for downstream analyses. CtGAP has been successfully used to assemble and characterize *Ct* genomes, perform *ompA* genotyping, genomic backbone and multi-locus sequence typing (MLST), plasmid detection, and phylogenomic analysis (2).

**Fig 1:**
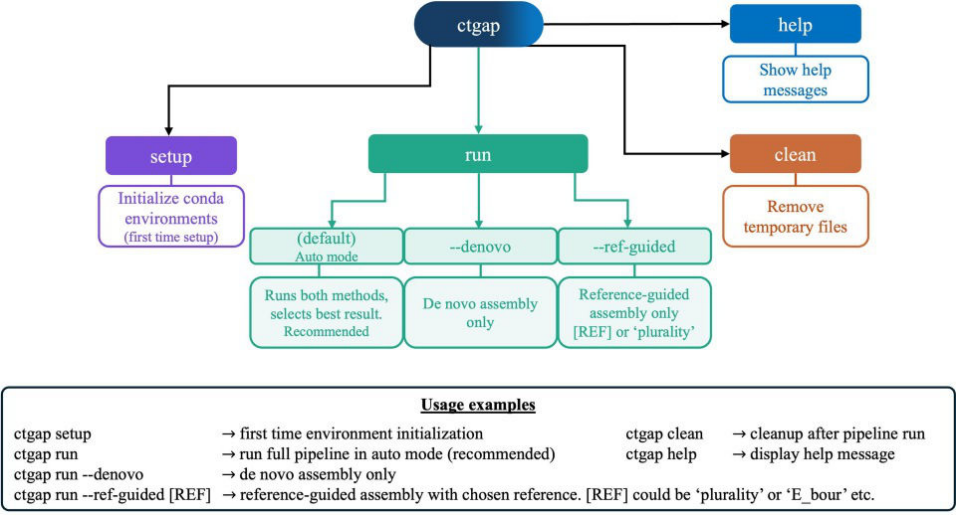
CtGAP 2.0 command-line interface. [REF] can be replaced with any reference genome name (e.g., E_bour) from the list of *Ct* reference strains available in “resources/references/ct.”

CtGAP 2.0 features significant architectural improvements and updated tools. Enhancements include a streamlined command-line interface that allows mode and reference selection (Fig. 1), a seamless installation process eliminating the need for multiple standalone tools, and an automatic assembly quality scoring and selection system that addresses a key challenge in bacterial genomics: determining which assembly method produces superior results for a given sample. When operated in “auto” mode, CtGAP 2.0 performs both *de novo* and reference-guided assembly, then automatically selects the optimal assembly based on a composite quality score incorporating N_50_, contig count, total assembly length, and GC content. This scoring system is calibrated for *Ct*’s expected genome characteristics (1.04 Mb chromosome, ~41% GC, ideally 1–2 contigs representing the chromosome and ~7.5 Mb plasmid). The best-performing assembly is automatically selected for all downstream analyses. The assembly mode framework has also been simplified. The original “both” mode, which executed *de novo* and reference-guided assembly methods and analyzed each independently, has been replaced with the “auto” mode, which eliminates downstream duplication and provides quality comparisons through a scoring framework. Users can still select either mode for specific use cases or make edits, if needed, to the scoring framework to suit their assembly needs. This architectural change required complete workflow modularization, improving maintainability and enabling future extensions.

Additional updates include integrated *Ct* strain typing via average nucleotide identity (ANI) and Mash distance calculations for comparing assembled genomes against a curated database of 26 *Ct* reference strains. The system employs a two-method consensus approach: fastANI provides high-resolution species-level comparisons (3), while Mash offers rapid k-mer-based distance estimates (4). CtGAP 2.0 also features an expanded *ompA* genotyping through a dual-step approach. Any genotype first identified as part of the ocular lineage undergoes an additional interrogation against an expanded set of manually curated *ompA* genotypes, enabling improved characterization of *ompA* diversity among lineage-specific strains. A new plasmid analysis module has been added, performing dedicated assembly and quality checks, BLAST analysis against a manually curated database of plasmid sequences, and MLST typing. Table 1 shows a summary of improved features now available in CtGAP 2.0.

**TABLE 1:** Comparison of CtGAP and CtGAP 2.0 showing improvements in key features as underlined

Feature	CtGAP	CtGAP 2.0^*a*^
Installation	Clone repo + download databases separately	Clone repo (all databases included)
Database	Manual download of host genome, Kraken2 DB, and *Ct* references	Pre-packaged with distribution
Setup	Manual conda env + path exports + dependencies build	ctgap setup (automated, one command)
User interface	snakemake -j n --use-conda -k	ctgap run (with intuitive flags)
Command-Line interface	Config file editing required	CLI flags: --denovo, --ref-guided, --auto
Assembly modes	denovo, reference-denovo, both	denovo, reference-denovo, and auto
Default mode	denovo (manual selection required)	auto (runs both, selects best)
Assembly selection	Not available	Automatic quality-based scoring (N50, contig count, length, and GC%)
Assembly scoring	Not available	Composite score (0–100 scale) with detailed metrics
Selection transparency	Not applicable	Per-sample reports with scores, metrics, and reasoning
Assembly filtering	Not available	minimap2-based contig filtering (removes non-Ct)
Gap reporting	Not available	Reports Ns in main contig
Workflow architecture	Mixed assembly and typing rules	Modular: separate assembly, selection, and typing modules
*Ct* Strain typing	Not available (separate pipeline required)	Integrated ANI + Mash (entire genome)
Mixed strain detection	Not available	Heterozygous site analysis (flags potential mixtures)
Genotyping	BLAST (*ompA*), MLST (plasmid, chromosome)	BLAST (*ompA*, plasmid), MLST (plasmid, chromosome)
Genome annotation	Not available	Bakta (coverage-gated at 90%)
Plasmid module	Not available	Comprehensive
Assembly reports	Per-mode QUAST reports	Per-mode + selection reasoning + summary statistics
Output organization	Flat structure	Organized: per-sample/, reports/, results/, phylogeny/
Reproducibility	Version-dependent (user downloads)	Fully reproducible (fixed database versions)
Documentation	README on GitHub	Comprehensive: README, help system, and inline documentation

^*a*^ Underlined text in column C denotes features that are new in CtGAP 2.0 compared to the prior version (column B).

CtGAP 2.0 is implemented in Python and distributed as a Snakemake workflow with full conda environment management and automatically handles dependency installation through conda/mamba. Installation instructions and documentation are available at https://github.com/D-Dean-Lab/CtGAP/tree/main.

## Data Availability

CtGAP 2.0 and all associated files are available at https://github.com/D-Dean-Lab/CtGAP/tree/main. The repository has installation scripts, test data sets, usage examples, and detailed documentation, including reference Ct genomic strain-typing databases, ompA genotyping, plasmid analysis, MLST schemes, and test data. CtGAP is open-source software under the MIT License at https://github.com/D-Dean-Lab/CtGAP (DOI: https://zenodo.org/records/18341706?utm_source).

## AFTER EPUB

*[This article was published on 24 February 2026 with an error in the affiliations. The affiliations were corrected in the current version, posted on 12 March 2026.]*

## EDITOR'S NOTE

**Ed. Note:** This article was handled by Pat Schloss, who acted as a Guest Editor in consultation with the Editor in Chief, Irene Newton. Dr. Schloss's role as the Guest Editor of this article is outside of his duties as the chair of the Journals Committee.
